# Biomimicry-Based Design of Underground Cold Storage Facilities: Energy Efficiency and Sustainability

**DOI:** 10.3390/biomimetics10020122

**Published:** 2025-02-18

**Authors:** Mugdha Kshirsagar, Sanjay Kulkarni, Ankush Kumar Meena, Danby Caetano D’costa, Aroushi Bhagwat, Md Irfanul Haque Siddiqui, Dan Dobrotă

**Affiliations:** 1 Department of Civil Engineering, Symbiosis Institute of Technology, Symbiosis International University, Near Lupin Research Park, Gram: Lavale, Tal: Mulshi, Pune 412115, Maharashtra, India; sanjay.kulkarni@sitpune.edu.in (S.K.);; 2 Department of Mechanical Engineering, College of Engineering, King Saud University, Riyadh 12372, Saudi Arabia; msiddiqui2.c@ksu.edu.sa; 3 Faculty of Engineering, Department of Industrial Engineering and Management, Lucian Blaga University of Sibiu, 550024 Sibiu, Romania

**Keywords:** biomimicry, underground cold-storage, energy conservation, sustainability

## Abstract

Underground cold storage gives rise to special challenges that require innovative solutions to ensure maximum energy efficiency. Conventional energy systems tend to be based on high energy use, so sustainable solutions are crucial. This study explores the novel idea of biomimetics and how it might be used in the planning and building of underground cold storage facilities as well as other infrastructure projects. Biomimetic strategies, inspired by termite mounds, gentoo penguin feathers, and beehive structures, are applied to minimize reliance on energy-intensive cooling systems. These natural models offer efficient thermal regulation, airflow optimization, and passive cooling mechanisms such as geothermal energy harvesting. The integration of naturally driven convection and ventilation ensures stable internal temperatures under varying conditions. Biomimicry was employed in Revit Architecture, coupled with structural optimization, to eliminate urban space’s limitations and further increase energy efficiency. The analytical work for this paper utilized a set of formulas that represent heat flow, thermal resistance, R-value, thermal transmittance, U-value, solar absorption, and G-value. The results pointed to very good insulation, with exterior walls having an R-value of 10.2 m^2^K/W and U-value of 0.98 W/m^2^K. Among the chosen 3-layer ETFE cushion with a U-value of 1.96 W/m^2^K, with a G-value of 0.50, showed good heat regulation and daylight management. Furthermore, bagasse-cement composites with a very low thermal conductivity of 0.10–0.30 W/m·K provided good insulation. This research proposes a scalable and sustainable approach in the design of underground cold storage by merging modelling based on Revit with thermal simulations. Biomimicry has been demonstrated to have the potential for changing subterranean infrastructure, conserving energy consumption, and creating eco-friendly construction practices.

## 1. Introduction

The escalating trajectory of urbanization, coupled with the surge in energy demand has made it difficult to build energy-saving buildings in limited spaces. An in-depth literature review on biomimicry, cold storage, and underground construction establishes the foundation for the study. Biomimicry is the idea of using nature’s design and environment to inspire the design of the project which is more energy efficient and solves the problem of the city growing in the horizontal direction. Therefore, the construction sector plays an important part in meeting the needs and requirements of the world [1]. The demand for energy-efficient cold storage facilities to preserve fresh produce over an extended period has led to the exploration of innovative design approaches. One such approach is biomimicry, which draws inspiration from nature’s ingenious designs to create sustainable and efficient systems. Rising affluence in major developing countries (principally China and India) and increasing diversion of agricultural resources for energy production (USA and Brazil) sharply increase agricultural resource demand [2]. Mankind has often taken inspiration from nature to solve problems since nature has sophisticated processes—redefined for thousands of years. An innovative way through which urban planning and disaster-resistant infrastructure could be approached is provided by biomimetic design principles that grasp the resilience and adaptability of nature for enhancing sustainability. While many manmade systems are unsustainable, natural processes embody sustainability principles; therefore, there are many things to learn from nature to solve design problems and create a more sustainable future [3]. Around 33.1% of total production is wasted each year due to the ineffective and inefficient post-harvest stages of the supply chain in India [4]. As of 2023, the cold storage market in India is valued at approximately INR 2052.7 billion (USD 24.9 billion). The market has been experiencing rapid growth driven by increased demand for temperature-sensitive products in sectors such as pharmaceuticals, healthcare, agriculture, horticulture, processed food, and dairy products. There are currently 6227 cold storage facilities in India with a storage capacity of up to 30 million tons [5]. A cold storage unit comes into the picture for this purpose, but the traditional method is having a place with a full refrigeration system which not only occupies a lot of space but also consumes a huge amount of electricity. Traditional techniques of underground cold storage desired thermal performance by using insulation materials, mechanical cooling, and passive ventilation. Although these methods have been applied in the past, are still being applied today, and are still relatively cheap, they may not be as energy-efficient or sustainable as biomimetic designs. And therein lies the other innovative alternative to the refrigeration system that reduces mechanical cooling dependency: the biomimetic method of building inspired by nature’s effective heat gain and cooling strategies, for example, penguin feather-inspired thermal resistance and honeycomb-like modular organization. However, proper refinement that fosters increasing availability through long-term performance monitoring would greatly help validate these concepts.

In recent years, with focus shifting towards energy-efficient measures designed for underground cold storage systems, it appears that many of the extant solutions fail to offer the requisite level of energy efficiency. Traditional methods such as insulation and active cooling all require considerable energy inputs and are thus not very adaptable to different thermal loads. Furthermore, they completely ignore taking advantage of the natural properties afforded by the surroundings, which is critical for enhancing energy efficiency in underground applications. Because of these limitations, the need arises strongly for novel strategies directed toward the resolution of such unique challenges. At this stage, biomimetics provides a way forward. Looking at it from a biomimetic perspective allows one to embrace natural strategies in improving energy efficiency of underground cold storage. Termite mounds, for instance, are a great template in achieving passive ventilation and temperature regulation without drawing on any externally supplied energy. Desert plants provide the paradigm to develop solutions to control thermal fluctuation in underground environments.

Thus, an attempt is made to substantiate the divide left between the conventional energy-saving methods and the innovative biomimetic solutions. The study establishes a unique, wide-ranging framework for the incorporation of nature-inspired strategies into underground cold storage schemes, showing their viability and efficiency through quantitative calculations and computational simulation. In the end, the article assesses scalability and adaptability, thereby creating room for an academic and real-life investigation of these strategies. The studied approaches modify the setting exceptionally while providing an eco-friendly approach for this particular research area.

Despite the growth, the number of cold storage in India is still inadequate as compared to the requirement. Currently in the country, cold storage is available only for single commodity potatoes, oranges, apples, grapes, pomegranates, flowers, etc., which results in poor capacity utilization. In contrast, underground cold storages have benefits such as the constant temperature being maintained because of soil (20–30 feet below the ground), which allows for the storage of produce year-round for a fraction of energy use, protection from elements, and minimal disruptions to the basements and foundation. Cold storage plays a crucial role in enhancing sustainability and supporting the United Nations Sustainable Development Goals (SDGs). Effective cold storage systems help address several SDGs by reducing food waste, improving food security, and promoting sustainable consumption and production patterns. According to SDG 9 that speaks about the industry, innovation and infrastructure investing in modern, energy-efficient cold storage infrastructure supports sustainable industrialization and fosters innovation. This leads to a more resilient supply chain and promoting economic growth while minimizing environmental impact.

The application of “Life’s Principles” from biomimicry would enable infrastructure managers to contend with complexity and uncertainty better; however, current practices appear to largely violate these principles [6]. Biomimicry engenders collaborative design tactics for urban environments and enhances smart city paradigms [7]. In its bid to operationalize biomimetic design, researchers have proposed avenues such as pre-emptive biomimetic research thoroughfare for multiple projects and the introduction of ecological performance standards in environmental impact assessments and sustainability ratings [8]. The avenue would provide the way toward addressing place-based resilience and adaptive design, which entails favourable environmental outcomes while addressing the complicated 21st-century predicament [8].

## 2. Materials and Methods

**Figure 1:**
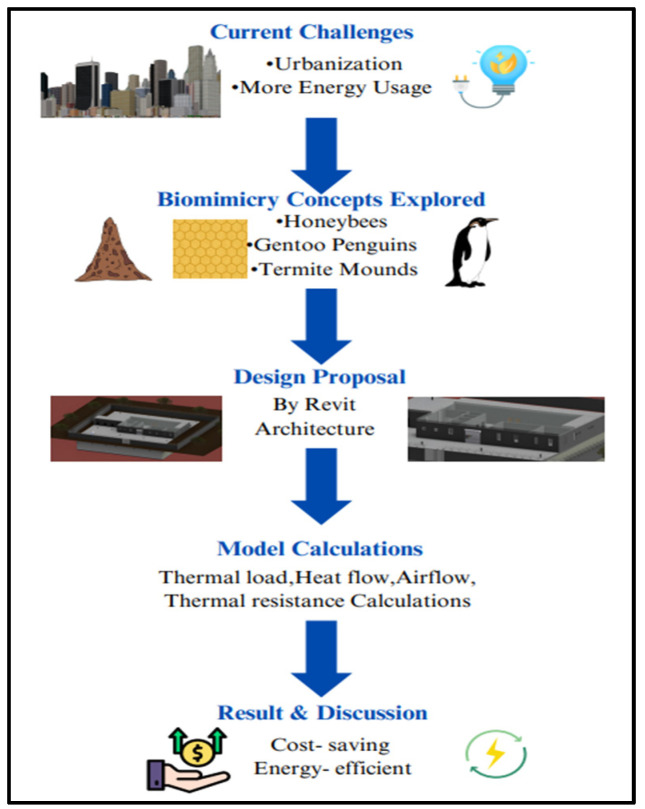
Methodology Flowchart.

This section covers the different aspects of biomimicry used in the project while discussing the traditional cold storage’s limitations and the facilities needed to function properly. This also discusses the underground development and how this is the future of the upcoming civil engineers with the thermal load calculations. Figure 1 shows the flow of the present work.

### 2.1. Biomimicry

Biomimicry in architecture can enhance sustainability and efficiency. Termite mounds inspire natural ventilation systems, reducing energy use. The layout of this lattice is like a network of tunnels that can intercept wind around the termite mound to create turbulence inside which proper ventilation and control of the interior climate can be taken care of. Iceberg structures save land, promoting vertical construction. The overall concept of cold storage is based on the imitation of iceberg structure; only a small proportion of the structure is visible on the surface, and the rest is beneath the surface. Bees’ hexagonal hive design is mathematically proven to have maximum storage capacity. The use of this concept to increase the overall storage efficiency and reduce the raw materials to be used. Penguin feathers’ insulation properties are emulated in building materials to maintain ambient temperatures and minimize heat loss. These nature-inspired concepts contribute to eco-friendly and energy-efficient building design.

### 2.2. Cold Storage

By 2050, the global population is expected to surmount 10 billion, necessitating a 70% surge in food production [9]. A cold storage facility is a complex thermal system that works for the preservation and efficient utilization of perishable food commodities [2]. A cold storage warehouse is a specialized facility equipped with temperature-controlled environments, primarily designed to store temperature-sensitive products. These products often include perishable goods like fresh produce, frozen foods, and pharmaceuticals. Completely functional cold storage has features like easy access for transportation (ensuring smooth entry and exit points facilities for seamless movement of goods in and out of the storage), sufficient storage capacity to meet your needs (having adequate spacing ensures that perishable goods can be stored efficiently without overcrowding), efficient inventory management (they should have systems in place to track products, manage stock levels, and rotate items based on their life-span), reliable power (uninterrupted power supply is essential for maintaining the desired temperature within the facility), and lastly, security. After analyzing all these essential elements and assessing various locations, the construction site for the project can be determined. Subsequently, a more detailed investigation of the site is necessary to understand the soil type, rock composition, and overall geological context. Another critical consideration when choosing the soil type is ensuring that it can withstand the excavation process effectively. While biomimicry shows great potential, its widespread adoption faces obstacles such as high initial construction costs, maintenance complexities, and lack of expertise [10]. Limited awareness and education among stakeholders also hinder its implementation [11]. Despite these challenges, biomimicry continues to inspire technological advancements, with some human-made systems even surpassing their natural counterparts in certain aspects [12]. Increased training, collaboration, and research are needed to fully harness biomimicry’s potential in sustainable urban design.

### 2.3. Underground Development

The constant population growth has led to land scarcity, urban crowding, and complex city planning. The orthodox methods of planning a city are no longer suitable. Underground construction is often seen as a desperate option rather than a smart one by planners. Constructing underground homes or facilities would have a significantly lower maintenance cost when compared to a conventional surface structure.

The main advantage of underground homes is that they are fire-resistant and energy-saving and would furnish safe living conditions in extreme conditions. Earth-ship homes are homes that are built of natural and recycled materials. The usage of materials is much less than in customary houses. While there are many advantages, the problems that arise while constructing underground structures are many. If the design and construction are poor, then it could lead to terrible ventilation and daylight, respectively. Another set of issues is related to natural calamities, i.e., flooding and collapsing. Last but most important is the attack of insects, rodents, humidity, darkness, etc., which might affect the quality of food. It must be noted that the initial construction cost of these projects is significantly higher compared to surface structures, because of the use of advanced machinery and the requirement of skilled labour [13].

Underground storage systems enhance energy and food security but cause additional environmental problems like groundwater contamination, flooding, and soil erosion. Thermal energy storage may influence the groundwater and thus warrants strict regulation [14]. To mitigate these issues additional biomimicry features like support provided by biomimetic root-inspired foundations will provide good resilience to flooding and erosion [15]. Caves of carbonate, like Magellan Cave in Armenia, are ambient for food storage [16] and can be mimicked. Risks will be negated by implementing additional biomimetic techniques of stabilization like live staking, crib walls, etc., [17]. This strengthening of soil with MICP minimizes disruption to the groundwater [18,19]. Scaling up MICP and biomimetic techniques will fortify subterranean infrastructure against environmental risks. However, further research is needed to standardize and upscale these biocementation techniques for widespread application in erosion control and soil stabilization.

In projects like these, thermal load calculation is the most important parameter that needs to be studied before designing and construction. Considering the losses that occur due to energy flux from the external environment, the thermal exchange from the stored products, lighting and ventilation systems, and thermal loads derived from the personnel, the thermal loads can be calculated using the formula as in Equation (1):

Q = k × A × ΔT(1)

where Q: Amount of heat transferred per unit of time.

k: Thermal conductivity.

A: Cross-sectional Area of the heat flow.

ΔT: The temperature difference between two ends of the material.

Underground thermal energy storage systems, including cold storage, are influenced by various factors that affect their performance. The equation Q = k × A × ΔT is limited for non-stationary temperature situations, as underground temperatures vary seasonally and with depth [20]. Soil temperature and heat exchange in cold storage are impacted by floor location and environmental conditions, requiring non-stationary analysis methods [21]. Convection heat transfer, driven by groundwater and airflows, can significantly affect thermal energy storage efficiency in underground systems [22]. The one-dimensional heat equation can be extended to include the effects of rainwater flow, which influences subsurface temperature propagation [23]. These factors highlight underground thermal systems’ complexity and simplified equations’ limitations. To improve the accuracy of heat transfer analyses, researchers should consider implementing more comprehensive models that account for dynamic conditions and real-world complexities. Additionally, clearly stating modelling assumptions, limitations, and their potential impact on conclusions is essential for transparent and reliable research [24].

The underground cold storage facility has a low thermal load due to no sun radiation, low temperature, and good insulation. It also saves more energy and money and pays off the high building cost. The best result is that the underground facility uses 60–70% less heat than the surface one with the same structure. In order to ensure proper storage in a cold storage facility, all must produce be of good quality. Even a few degraded can have a negative impact on the entire contents of the package or the cold storage itself. It is essential that commodities can maintain a consistent temperature and relative humidity. Proper handling practices include temperature management, relative humidity control, air circulation, and maintaining adequate space between containers for proper ventilation. It is also important to avoid incompatible product mixes.

## 3. Location, Layout, and Design

The primary consideration when choosing a location for a cold storage facility should be its intended use. Additionally, any cooling and packing facilities should be situated in close proximity to fields and orchards to minimize the time between harvesting and the start of the cooling process. When designing a cold storage, it is essential to take into consideration various factors such as temperature, relative humidity, rainfall, wind, and water. These factors play a crucial role in ensuring the proper functioning of the facility. Irrespective of the location of the facility, the cold storage must comprise chambers that are spacious enough to meet the storage demands. Additionally, a pre-cooling room and a packing, grading, and sorting area must be strategically placed for the effective functioning of the cold storage facility. The site selection must be made considering the availability of adequate energy and water supply. The walls, ceilings, and floors of the cold storage must be well-insulated to maintain the desired temperature. Dispatch and receiving docks must be provided adequately, keeping in mind the size of the vehicles and parking space requirements.

After excavation work in all underground construction, a retaining wall is provided on all sides of the structure. This is to prevent soil erosion, preventing soil from sliding onto the structure. Before the design phase, it is crucial to consider the details about the properties of the produce being stored, the regularity of unloading and loading, harvesting seasons, and the “best-before” dates of all the produce. These details will aid in designing a cold storage facility that meets the storage demands effectively.

From the above the discussion, it can be concluded for cold storage facility following parameters need to be checked:

1. The property of the produce being stored
2. Regularity of unloading and loading
3. Harvesting seasons and “best-before” dates of all the produce
4. Field heat of the produce
5. The daily production capacity
6. The expenses associated with maintenance, electricity, and other relevant costs

### 3.1. Capacity

Capacity refers to the maximum amount of good that can be stored or accommodated [6] and here it is measured in metric tonne (MT).

Calculation of storage capacity in the present study:

- Area of cold storage chamber = 28 × 15 = 420 m^2^
- Maximum stacking height = 3 m
- Hence, the maximum volume of produce that can be stored = 420 × 3 = 1260 m^3^
- Assume the average density of all the produce to be 850 kg/m
- Hence, the volume required for 1 MT (1000 kg) = 1000/850 = 1.2 m^3^/MT
- Therefore, the storage capacity of 1 cold storage chamber = 1260/1.2 = 1050 MT
- Area of pre-cooling room = 20 × 14 = 280 m^2^
- Maximum volume of produce that can be stored = 280 × 3 = 840 m^3^
- Volume required for 1 MT (1000 kg) = 1.2 m^3^/MT

Therefore, the storage capacity of 1 pre-cooling room = 800/1.2 = 700 MT.

Hence, the total storage capacity of the cold storage facility: (1050 × 2) + (700 × 2) = 3500 MT.

The design storage capacity for the proposed underground cold storage is 3500 MT. Two cold storage chambers, each with a capacity of 1050 MT will be provided at each level of the subsurface facility, along with two pre-cooling rooms, each with a capacity of 700 MT.

### 3.2. Gross Volume and Total Volume of Cold Storage

The gross volume of a cold storage capacity is the complete volume of the facility that can contain all the produce and goods, excluding unused areas not meant for storage. The calculation of gross volume and storage capacity differ, as the former considers the total height of the storage space, rather than the maximum height of stacking [25].

Calculation of gross volume:

- Total volume of cold storage chambers = 28 × 15 × 4 = 1680 m^3^
- Total volume of pre-cooling rooms = 20 × 14 × 4 = 1120 m^3^
- Total volume of grading, sorting and packing area = 28 × 19 × 4 = 2128 m^3^

Hence, gross volume of the cold storage facility: (1680 × 2) + (1120 × 2) + (2128 × 2) = 9856 m^3^.

The total volume may be defined as the entire volume of the cold storage facility, including the space within all the walls, slabs, and other structural members of the facility [25].

Calculation of total volume:

- Total volume of surface facility = [(14.6 × 4.5) + (29 × 12)] × 4.5 = 1861.65 m^3^
- Total volume of the subsurface facility = (43.5 × 45 × 9) = 17617.5 m^3^

Hence, total volume of the cold storage facility: 1861.65 + 17617.5 = 19479 m^3^.

The ratio of gross volume and total volume gives an index of how well the cold storage facility has been designed. The ratio must be in the range of 0.80 to 0.50 [25]. For the proposed cold storage, the ratio is found to be 0.505. Hence, the ratio is just within the range.

## 4. Requirements for Construction of Cold Storage

A cold storage has some basic requirements before the actual the construction starts. Therefore, this section deals with the temperature requirements, the type of produce that can be stored, and the heat load on the facility.

### 4.1. Temperature Requirement

Designing a cold storage facility requires focused consideration of a temperature-controlled environment. Adequate insulation in the walls and slabs is essential to regulate internal temperatures and minimize heat loss. In order to achieve this goal, it is of utmost importance to carefully choose the suitable insulating materials that possess a high R-value and thermal mass. Maintaining the optimal temperature brings about numerous advantages, such as reducing the respiration rate of perishable goods, inhibiting enzymatic degradation, impeding the growth of microorganisms, and averting the production of ethylene, a natural ripening agent. In the case of underground cold storage facilities, an appropriate range of temperature is from −5 to 10 °C concerning ambient temperature and the type, as well as the variety, of stored products. Moisture content within the cooling chambers should be regulated so that it does not adversely affect the post-harvest cooling process; this necessitates the proper management of relative humidity inside these chambers throughout. As per the Food Safety and Inspection Service (FSIS), optimum conditions should be between 0 and 10 °C and have a relative humidity of 80–95%.

### 4.2. Type of Produce Stored and Heat Load

The type of produce that can be stored in any cold storage structure depends on three main factors—Internal Temperature, Relative Humidity, and shelf life of produce. At room temperature, a large number of fruits and vegetables have a very low shelf life which makes it extremely important to store them in pre-cooling rooms, where post-harvest cooling takes place. For any cold storage to have a variety of produce, a flexible temperature range has to be selected and economical. The storage of perishable produce in cold storage has improved in the last few years which has, in turn, resulted in greater organoleptic quality preservation, less spoilage, and much longer shelf life. When storing produce, especially in multi-produce cold storage, utmost care must be taken to store only those products which are compatible with each other [25]. Maintaining a constant temperature is the key to attaining the maximum advantage of cold storage. Heat load which is the total amount of a refrigeration system that must be removed from the cold storage facility [25].

## 5. Model Calculations

**Figure 2:**
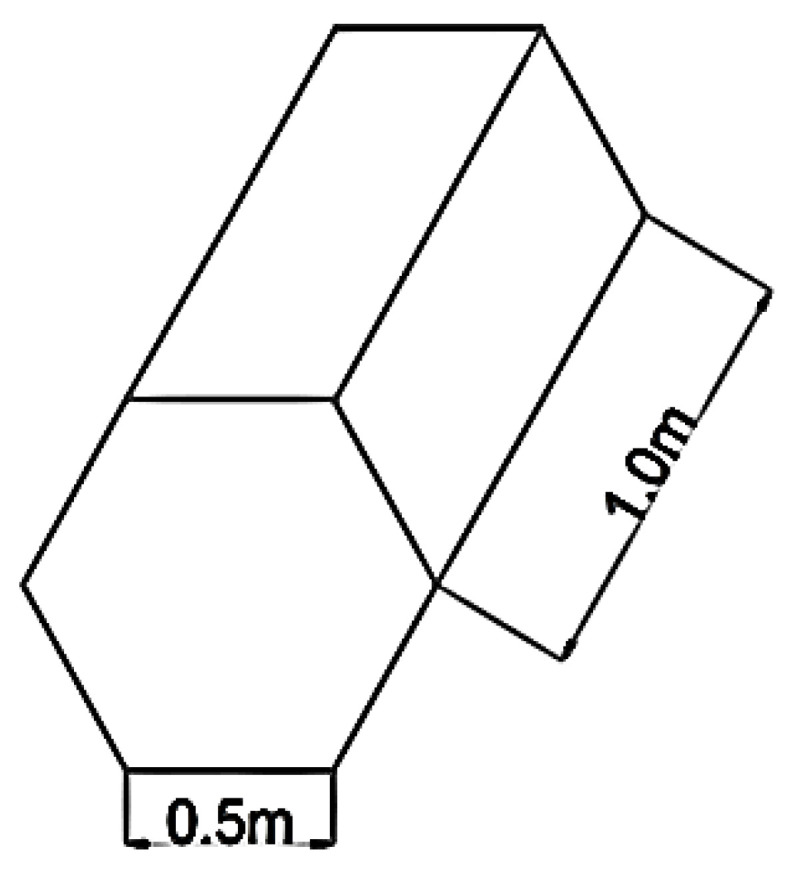
Dimensions of the proposed storage boxes.

This section deals with the design of the storage boxes its volume of each box as well as the volume occupied by 100 tonnes of onions and the wood used per box. It also discussed the heat transfer through the walls and the formula for the same. Figure 2 Shows the dimensions of the proposed storage boxes.

### 5.1. Design of Storage Boxes

- Volume of the total product = Total weight of produce/Bulk weight of produce = 100,000/970 = 103 m^3^
- Assumed length of one side of the hexagon = a = 0.50 m
- Assumed height of each box = h = 1.0 m
- Therefore, volume of each box = (3 × √3/2) × a^2^ × h = 0.65 m^3^
- Bulk density of wood used for storing the boxes = 850 kg/m^3^
- Weight of produce in each box = (Volume of each box) × (Bulk density of wood) = 0.65 × 850 = 552.5 kg/box
- Total number of boxes required = (Total weight of produce)/(Weight of produce in each box) = 100,000/552.5 = 181 boxes
- Thickness of each box = t = 0.005 m^3^ all around
- Actual volume of wood used per box = 0.026 m^3^ per box
- Total volume of boxes = (Volume of wood used per box) × (Total number of boxes) = 0.026 × 181 = 4.7 m^3^
- Total Volume Occupied by 100 Tonnes of Onions = (103 + 4.7) = 107.7 m^3^

### 5.2. Heat Transfer Through Walls

For steady-state flow, the heat flow is given by [26]

Q = UA (To − T_i_) Kcal/h (2)

where

- U = overall heat transfer coefficient (Kcal/m^2^ h) = 1/(R-value)
- A = Surface area through which heat is transferred (m^2^)
- T_o_ = Temperature of outside air (°C)
- T_i_ = Temperature of inside storage space (°C)

For best heat transfer results, the R-value (Thermal Resistance) must be high, and the U-value (Thermal Transmittance) must be kept significantly lower. Thermal mass is the property of an element to store heat. The higher the thermal mass, the lesser the temperature fluctuations within a structure.

## 6. Application of Biomimicry Features for the Design of Cold Storage

In this section, various application of biomimicry features which are used in the design of the cold storage facility have been discussed.

### 6.1. Iceberg

**Figure 3:**
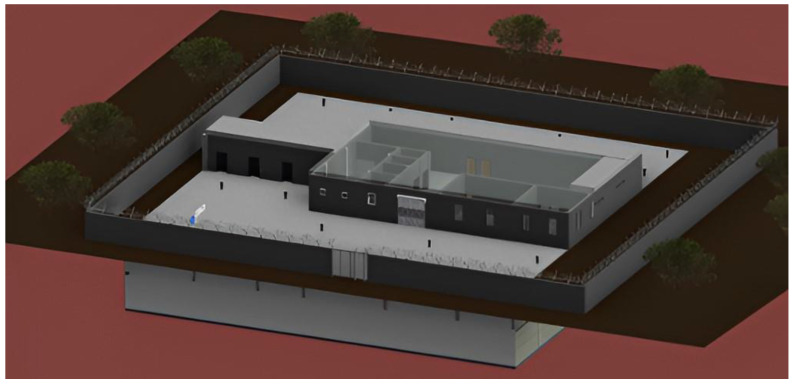
Iceberg concept implemented for the cold storage facility with (90:10) underground-to-above-ground ratio (made using Revit).

The underground structure uses the concept of iceberg where 90% of the structure lies beneath the ground and only 10% lies above the surface [9]. An iceberg optimizes the space where surface land is scarce and is very expensive. With the constant increase in population, decrease in the availability of land, and skyrocketing prices, the availability of land is going to be an important factor in planning and constructing future structures. While keeping all of this in mind, the project uses a similar concept where only one-fifth of the structure lies above the surface and is visible. Figure 3 shows the implementation of iceberg concept for cold storage design using Revit. Further detailed plans are attached as a Supplementary Material.

The two aims have been completed by using the concept of iceberg structures. They are as follows:

1. The design will make efficient use of the available underground space for construction.
2. It will make use of the natural insulating property of the underground environment to significantly cut down the energy costs which are typically higher for industries like cold storage and warehouses.

### 6.2. ETFE Roofing

**Figure 4:**
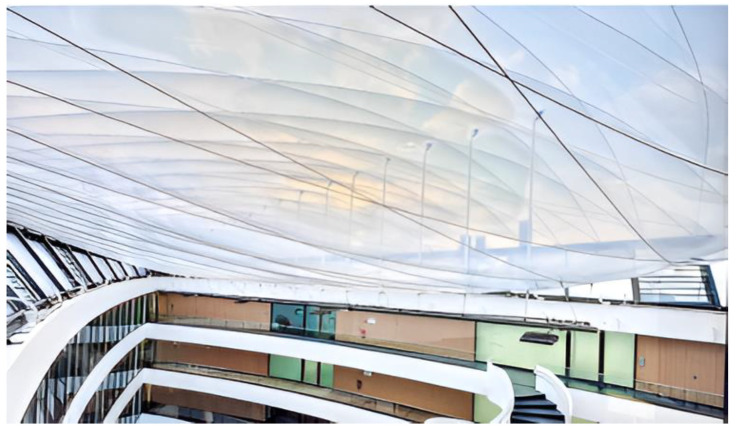
ETFE roofing in a research building in Braunschweig, Germany. Source: https://specialtyfabricsreview.com/wp-content/uploads/sites/28/2018/03/6907_20171222_1N4V9587_1_Hanno-Keppel.jpg (accessed on 1 December 2024). Photos: Hanno Keppel.

**Figure 5:**
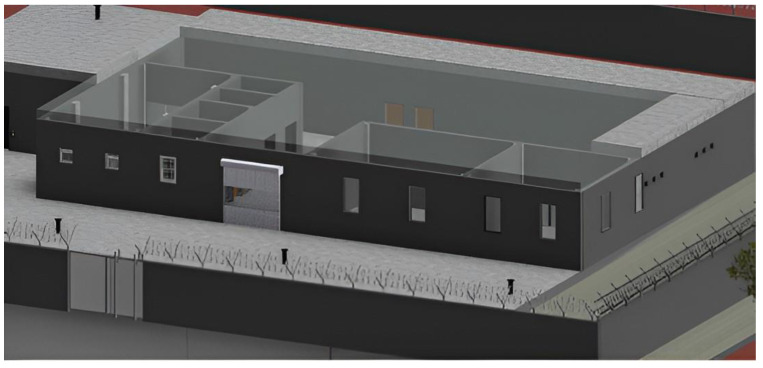
3-layer ETFE cushion system implemented in the surface warehouse facility (made using Revit).

ETFF is a light, rugged material that has become very common in roofing. It can take 400 times its weight without breaking, can stretch up to three times its length without losing its elasticity, and is repairable using tape patches. It also boasts a mud- and bird-repellent coat, a non-stick surface, and up to a 50-year lifespan, making it highly resistant to tears, offering excellent daylight transmittance, low energy consumption, and low maintenance [27] as shown in Figure 4. Locker rooms and restrooms will have solid concrete slabs to provide a private area, while for the cold storage warehouse, most of the structure will be covered using ETFE roofing, as designed in Figure 5 (Revit modelled). While ETFE offers cost savings and structural benefits due to its low weight, its thermal behaviour and impact on energy performance require further investigation [28]. The heat transfers through ETFE membranes, including heat loss and solar gains, affect a building’s energy use and interior conditions [29]. ETFE film systems, used in various configurations, significantly influence the climatic conditions and comfort of enclosed spaces [30]. This increased heat exposure is primarily due to reduced radiant heat loss and natural ventilation caused by the ETFE foil cushions [31].

### 6.3. Penguin Feathers

**Figure 6:**
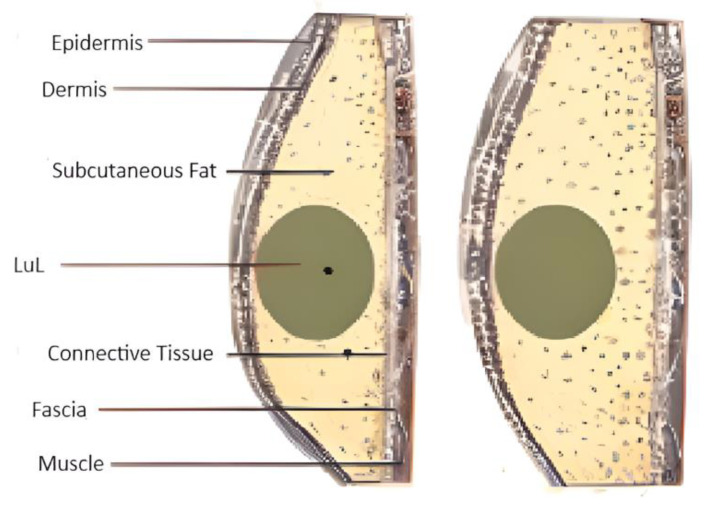
Body structure of a Gentoo penguin.

The Antarctic is very famous for its extreme conditions which make any sort of vegetation difficult to flourish. Gentoo penguins have very fine feathers that help trap the air and three layers that allow them to survive in freezing temperatures and their body temperature is constantly maintained at 32 °C, as shown in Figure 6. Creatures melt, changing their pelt or feathers completely when the old coverings are worn and tatty, depriving them of insulation and leaving them vulnerable to the cold. But Gentoo penguins (*Pygoscelis Papua*) moult in style. They do not slough off the old before growing the new. Instead, Gentoo’s keep holds of their old feathers, becoming super fluffy as the new ones emerge from beneath, doubling their insulation [32]. Similarly, the walls have the subsurface cold storage have three primary layers of insulation, which assists the structure in maintaining temperature and reducing thermal losses from the walls and slabs. The wall design includes 3% bagasse fibres, a sugarcane residue, mixed with the concrete which reduces the measured thermal conductivity from 0.62 to 0.46 W/mK [33].

**Figure 7:**
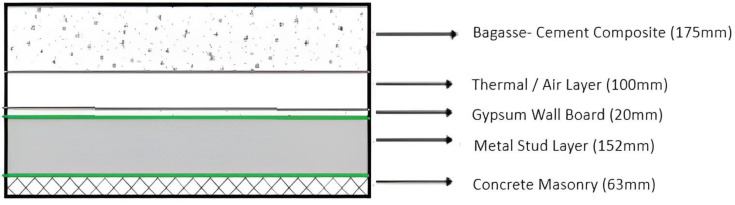
Cross-section of the exterior wall of subsurface cold storage facility showing the layers of insulation.

The final structural combination for the wall, as shown in Figure 7, increases the thermal resistance between the inside and outside of the cold storage, reduces energy transmittance and losses, and assists in maintaining a constant low U value, and a high thermal mass, thus increasing the cold storage’s overall thermal efficiency.

### 6.4. Termite Mounds

**Figure 8:**
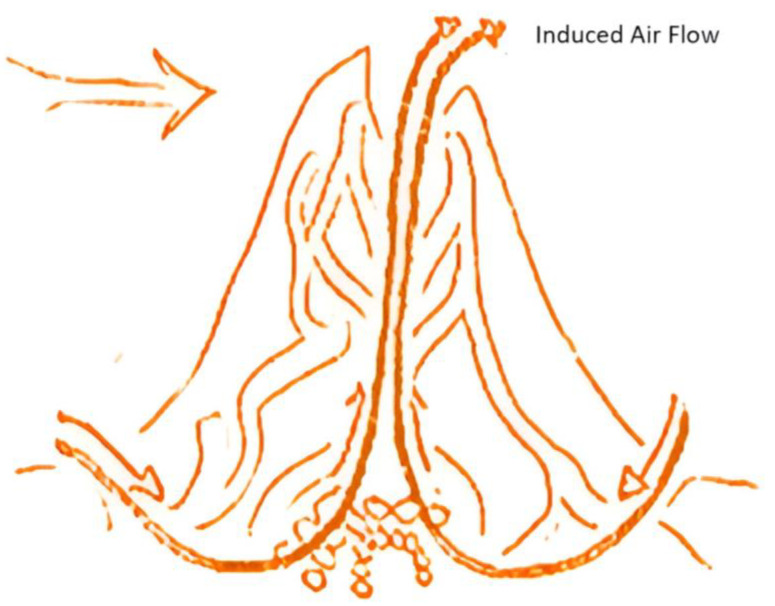
Induced flow model for termite mound ventilation.

Natural ventilation has been inspired by termite mounds not just in this project but also in high-rise buildings. The main idea behind the concept is that a termite mound ventilates itself and controls the internal and external climate by natural ventilation. A termite mound has single or multiple vents which help in ventilation, while others have no visible vents. The mounds’ designs range from cones to pillars to hemispheres. The most common use of this concept in commercial buildings is the induced flow ventilation method, better known as stack ventilation. It is created by a pressure differential between the outside and inside air of a system, or a structure, as a result of temperature variations. Because a termite mound extends upwards, as shown in Figure 8, the ‘vent’ is exposed to higher wind velocities compared to openings in the ground. Then, fresh air is drawn into the mound, circulates throughout the entire nest, and is finally dispelled through the opening of the mound.

The ventilation system that has been adopted for underground cold storage is a hybrid system. When natural wind speeds are inadequate, the system will surely fail and this calls for the need for mechanical ventilation which only operates when needed. The main driving forces for this system to work would naturally be the wind speed and temperature differences between the internal and external regions.

**Figure 9:**
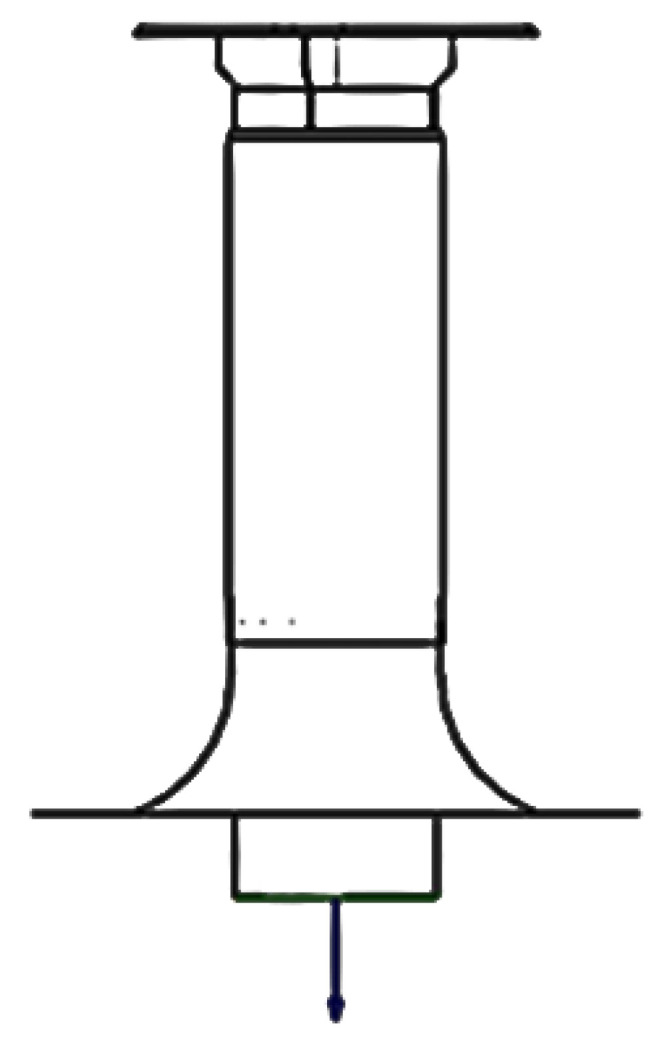
Schematic Drawing of ventilator hoods.

Ventilation hoods, as shown in Figure 9, are provided in the ground level slab that would facilitate easy natural ventilation of the G-1 level of the cold storage facility. After a series of diameter opening combinations, it was found that the 400 mm openings for the ventilator hoods would give the best ventilation results as shown in Figure 8 for schematic drawing. Therefore, 20 ventilator hoods with diameters of 400 mm would be provided on the ground floor slab. The main driving forces for this system to work would naturally be the wind speed and temperature differences between the internal and external regions.

### 6.5. Hexagonal Beehive Design

Hexagons have been proven, scientifically, to be the most efficient packing shape. The honeycomb is made up of prismatic hexagonal cells that are built by bees. To minimize surface area for a given volume and ensure smooth alignment of back-to-back cell ends, its back side forms a trihedral made up of three flat surfaces. This optimization preserves building materials (beeswax) while maximizing interior space and also being energy efficient. Because each hexagon cell’s shared walls equally transfer downward forces, hexagons are perfect for building beehives. This even dispersion strengthens the honeycomb’s robustness, which is a feature that is highlighted for its longevity. In a similar way, produce in the subsurface facility can be stored in hexagonal storage boxes, where each box represents a single cell of the honeycomb. When the boxes are placed in a honeycomb-like pattern, the storage efficiency of the subsurface cold storage would significantly increase. This would allow for the storage of a larger number of hexagonal boxes.

## 7. Results and Analysis

As discussed in earlier sections, there are applications of biomimicry for various parts of cold storage because of its design. This research contributes to the adoption of biomimetic methods by the more integrated approaches into a synergistic design for underground cold storage, which has been modelled and analyzed in Revit. Termite mound passive ventilation, iceberg-inspired thermal mass utilization, and beehive-inspired modularity have been incorporated; therefore, a unique agenda has been proposed for developing environmentally friendly refrigerated storage alternative techniques. This also emphasizes applying energy simulation methods to study the thermal performance of underground cold storage buildings. For analysis, thermal load, heat flow, airflow and thermal resistance formulas were used to assess parameters such as U-values, R-values, and solar energy absorption. For structural designing, Revit architecture was used. It is an extremely useful tool, especially when integrating biomimetic principles. Exterior walls show an R value of 10.2 m^2^K/W and a U-value of 0.98 W/m^2^K, exhibiting high insulation efficiency. The selected 3-layer ETFE cushion with a U-value of 1.96 W/m^2^K and G value of 0.50 reflects its capability to suppress heat transfer while facilitating controlled daylight transmission. This section discusses the design calculations and their effectiveness.

### 7.1. Analysis of Incorporating Iceberg Structure

There are two features discussed in this section about surface to subsurface ratio and land-use efficiency based on incorporating iceberg structure. The surface area to subsurface volume ratio, a comparison of land-use efficiency to surface cold storage, an analysis of the expenses associated with surface and subsurface cold storage systems, and analogies to the structure of an iceberg are the primary criteria under investigation.

From the calculations made from Table 1, the surface-to-subsurface ratio of the cold storage facility is 1: 4.7, which can also be written as 17.5%: 82.5%.

The surface-to-subsurface ratio of icebergs is usually between 10% and 90%. Since the cold storage facility’s ratio is similar to what was seen there, it can be said that this cold storage is having an iceberg-like structure [34].

Another feature is to calculate the land-use efficiency of constructing an iceberg structure; a comparison is made between the designed cold storage and a general surface cold storage facility of 3500 MT capacity for the storage box, where the area is 1960 m^2^.

For constructing a 300 MT cold storage, an area of approx. 2 acres (approx. 8500 m^2^) is required. This area of land should contain all the necessary facilities, namely, a warehouse, 3–4 cold storage chambers of 500 MT each, a grading, sorting, and packing hall, receiving and dispatch docks, and a pre-cooling room.

Therefore, the area required for a conventional cold storage facility = 8500 m^2^.

By implementing the concept of iceberg constructions, the land-use efficiency of the proposed cold storage facility:

Therefore, (8500 m^2^ − 1960 m^2^) = 6540 m^2^ or 77% of the land area is saved.

### 7.2. Analysis of Incorporating ETFE Roofing

For the analysis of the ETFE roofing system, the main factors that need to be considered are the U-value, G-value, and load analysis. A comparison between ETFE and other conventional roofing materials is carried out in this section.

The U-value of a material may be defined as the rate of transfer of heat for a given structure [25]. A lower U-value is beneficial as this indicates that the material is a better insulator. Thus, the U-value indirectly reflects the insulating property of a material. The G-value of a material indicates how much solar heat may be transmitted through the material. If the G-value is low, it means that only a small percentage of solar heat is allowed to pass through the structure. Thus, the G-value indirectly affects the ambient conditions of a structure and the thermal comfort levels.

While it would be possible to adopt the 4-layer ETFE cushion due to a lower U-value, the cost of installation and maintenance is higher. Fritting may be performed to the 3-layer ETFE cushion, which means that a series of irregular patterns may be printed on the layers of the cushion which would help in achieving a better G-value by reflecting most of the solar heat. Table 2 shows the U-value and G-value of conventional roofing materials and the combinations discussed in this section.

In many projects, glazed glass is becoming a highly conventional roofing material. Because of its light weight and excellent light transmittance properties, many structural engineers prefer glass over concrete slabs in commercial buildings. However, with the revelation of ETFE as a roofing material, few countries have begun adopting this material in most of their structures.

- ETFE is a very versatile material and can easily be turned into a cushion as wide as 25 m × 3 m, whereas glass is very brittle and has small structural dimensions.
- ETFE layers are transparent in visible light and UV radiation ranges, allowing for around 95% and 85% transparency, respectively. The visible light transmittance of clear glass is 90%, and the UV transmittance is around 75%. Infrared light is absorbed very well by both.
- ETFE foils are unable to block longwave radiations. Glass, on the other hand, is able to block these radiations to an extent, depending on the thickness.
- ETFE is able to achieve lower values of thermal resistance with additional layers.

It is shown in Table 3 that the solar heat gain coefficient of ETFE is significantly higher, compared to glass. Thus, it is beneficial to have fritted or shaded ETFE cushions. The higher the frit density, the lower is the absorption of shortwave radiations. Since ETFE has a low weight, it reduces the overall structural weight. This means that the columns of the superstructure would have to bear less weight. Compared to glass, ETFE transmits more light, insulates better, and costs 24 to 70% less to install [36]. ETFE is only 1/100 the weight of glass, and it has properties that make it more flexible as a construction material and a medium for dynamic illumination.

The semi-transparent roofing system offered by ETFE has several advantages over opaque roofing, especially in applications like cold storage warehouses or structures that require controlled light conditions. The semi-transparent material allows natural light to pass through, and the artificial lighting can be used less during daylight hours, which again reduces energy consumption and improves the working environment. These roofs reduce artificially used light and, in turn, the power expenses on the electric bill. It also provides materials, like ETFE, that will allow a balance of daylight penetration and thermal control, hence highly insulating for reduced heat gain, which is essential in temperature-sensitive environments, such as cold storage. The aesthetic of such a semi-transparent roof also enhances a modern, open, light-filled design, visualizing an impressive building. Semi-transparent systems, different from opaque roofs that block natural light and increase lighting demands, allow for a balance between natural light exposure and energy-saving features, making them ideal for many sustainable building designs.

### 7.3. Analysis of Incorporating Gentoo Penguin Feather

The walls of the subsurface cold storage have three primary layers of insulation which assist the structure in maintaining temperature and reducing thermal losses from the walls and slabs. In this section, the R-value, U-value, and thermal mass of the designed external wall are analyzed and a comparison is also made between the designed wall and a wall with different structural combinations.

R-value is a heat property and a measurement of a temperature difference by which an object or material resists a heat flow. Thermal resistance is the reciprocal of thermal conductance.

Total thermal resistance (m^2^K/W).

R = Thickness/Thermal conductivity = t/λ(3)

**Figure 10:**
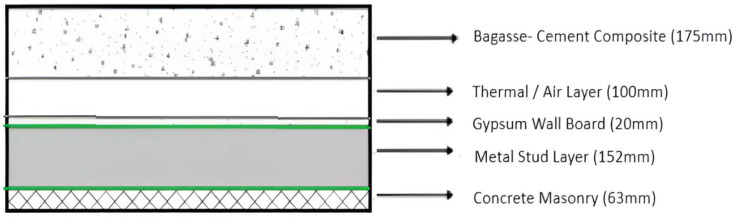
Structure A: Bagasse-Cement Composite Wall.

**Figure 11:**
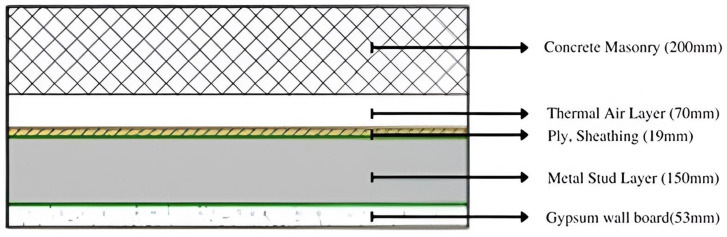
Structure B: Common insulating wall.

**Figure 12:**
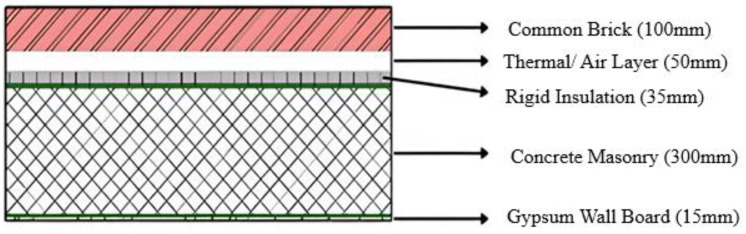
Structure C: Regular masonry wall.

It is necessary to compare the selected composite wall with a regular masonry wall and another wall of similar thickness but composed of different materials to prove its greater efficacy. The chosen wall is called Structure A in this instance, the typical insulating wall is called Structure B, and the traditional masonry wall is called Structure C, as shown in Figure 10, Figure 11 and Figure 12.

Using the above Equation (3), and Table 4, the thermal conductivity may be calculated as: 0.175/1.03 + 0.1/0.250 + 0.02/0.65 + 0.152/0.025 + 0.053/1.3.

Hence, the R-value for the exterior walls = 10.2 m^2^k/W.

The U-value of a material is a measure of how effective that material is as an insulator. U-value is also known as thermal transmittance and may be defined as the rate of heat transfer through any material or a composite structure. A low value indicates that the material is a good insulator and can minimize heat losses. The U-value of a material can be found by taking the reciprocal of the R-value.

U-value = 1/Thermal resistance = 1/R (W/m^2^K).

The R-value for the exterior walls = 10.2 m^2^k/W.

Therefore, U-value= 1/10.2= 0.098 W/m^2^K.

Table 5 below shows the U-value and G-value of the adopted 3-layer ETFE cushion compared to some other roofing materials.

Table 5 compares the various parameters used to select the structure for the exterior walls. Out of the three structures as shown in Figure 10, Figure 11 and Figure 12, it can be concluded that Structure A has the best results. In Structure A, there is a constant low U-value while also having a high R-value. It also has an excellent insulating ability and seepage control while having minimum heat loss. The only drawback is that the skill required for instalment is high.

### 7.4. Structural Stability of Incorporating Hexagonal Beehive Design

**Figure 13:**
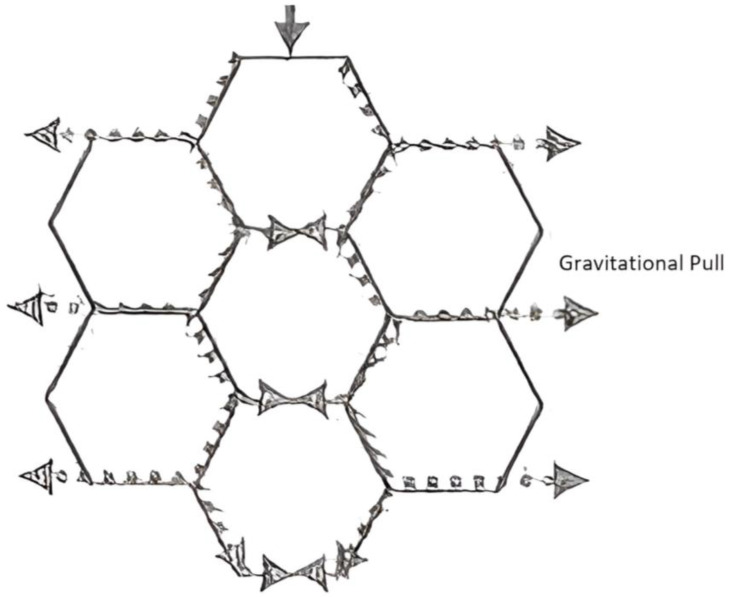
Spread of forces on a hexagon cell.

The produce that is stored in the underground cold storage is in hexagonal-shaped storage boxes. This results in the increase in the storage efficiency of cold storage, which in return, could allow a larger number of hexagonal storage boxes. They must be able to stand firmly on their own to avoid the downward force of gravity. Since each cell’s mutual walls mean that the structure’s downward physical stresses are spread equally, hexagons function well, as shown in Figure 13. As a result, they’re highly durable, and the force is uniformly spread around the racks and storage boxes.

**Figure 14:**
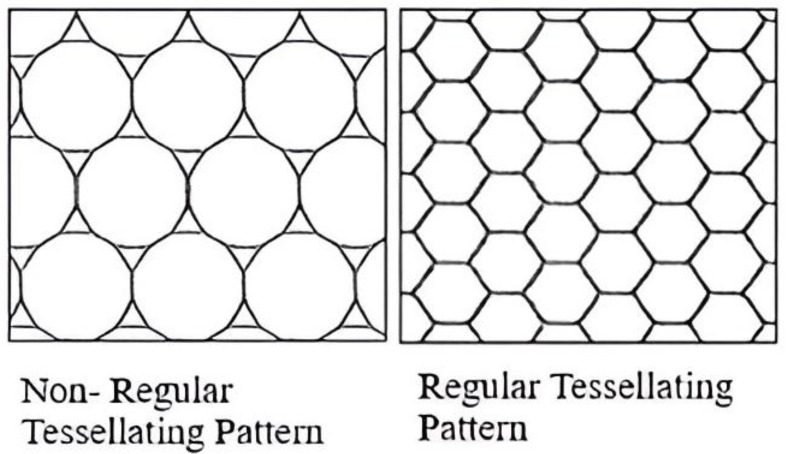
Difference between regular and non-regular tessellating patterns.

Honeycombs have perfectly formed tessellating patterns with no gaps or spaces. Since hexagons naturally produce regular tessellations, it is possible to generate tessellation patterns by simply replicating the shape itself, without the need for extra shapes to fill in any gaps. For a comparison of irregular and regular tessellation patterns, see Figure 14.

In polygons, more sides improve structural stability; in regular tessellating patterns, the converse is true. Patterns become regular when side numbers drop from erratic. Closed-pack hexagonal structures have a packing efficiency of 74%, which is higher than that of basic cubic (52%) and diamond (34%) structures. For this reason, hexagons are perfect for improving the cold storage facility’s durability and storage efficiency [37].

### 7.5. Analysis for Natural Ventilation

This part delves into the various types of ventilation (Natural, Mechanical and Hybrid) which was taken into the consideration while making some assumptions for easier calculations. Building fenestration can be responsible for a significant impact on the environment created in a building, affecting, either adversely or beneficially, both the health and perceptions of the occupants. An alternative to the traditional fenestration solution is ethylene tetrafluoroethylene (ETFE) [36].

For calculations, a few assumptions need to be made. They are as follows:

- Constant basic daily wind speed of 39 m/s. Assume that this wind speed does not fluctuate.
- The average temperature range of subsurface soil is between 10 and 15 °C.
- For calculation purposes, assume the mean value of 12.5 °C.
- The average internal temperature range of the subsurface cold storage facility is between −5 to 10 °C.
- For calculations, assume the mean value of 2.5 °C.
- Assume that the temperatures considered remain constant throughout the year.
- Assume that the wind blows in a direction that is perpendicular to the openings of the ventilator hoods.
- Assume that ventilation required for ambient conditions is 1.10 m^3/^min/m^2^.

The value E = 0.55 for perpendicular winds has an origin in experimental studies, both in wind tunnel and field experiments [38]. These studies show that turbulence, vortex formation, and pressure differences reduce airflow efficiency. The range of standard openings is generally between 0.5 and 0.6 [39]. Additional literature, including the ASHRAE Fundamentals Handbook (give ref), demonstrates consistent airflow coefficients for different geometries, confirming E = 0.55 as a good practical approximation. Hence, this assumption is considered. The rate of airflow is defined as the movement of air per unit time period. The airflow may be calculated using the Equation (4):

Q = E × A × V (m^3^/s) (4)

where

- Q is the rate of airflow E is the effectiveness of openings (taken as 0.55 for perpendicular wind direction)
- A is the free area of inlet openings (in m^2^)
- V is the wind velocity (in m/s)
- For the subsurface facility,
- 20 ventilator hoods of 400 mm diameter would be provided on the ground floor slab.
- Q for 1 ventilator hood = 0.55 × (π/4 × 0.42) × 39 = 2.70 m^3^/sec = 162 m^3^/min
- Total airflow rate = Q_total_ = 20 × 162 = 3240 m^3^/min
- Assuming 40% losses, Q_total_ = 60% × 3240 = 1944 m^3^/min

Hence, total ventilation achieved by natural means = 1944 m^3^/min.

Now, the area of the G-1 level of the cold storage, as calculated earlier, is equal to 1957.5 m^2^.

Then, Q_required_ = 1.10 × 1957.5 = 2153 m^3^/min.

Hence, total mechanical ventilation required = 209 m^3^/min.

By providing an optimum number of ventilation hoods of 400 mm radius openings, it is observed that very little mechanical ventilation is required. This would help to reduce the overall operational cost and increase the energy efficiency. The total amount of natural ventilation achieved by a set of 20 ventilator hoods with 400 mm diameter apertures is 1944 m³/min. The openings of each hood are 250 mm in diameter; however, this decreases to about 756 m³/min. This means that 1397 m³/min of mechanical ventilation—nearly seven times the capacity of the larger openings—is required. Therefore, choosing smaller diameters results in high running expenses.

Therefore, the ventilation hoods are designed in a compact manner to facilitate only a minimal amount of heat transfer and avoid convection and radiative heat transfer. Their design is well suited to restrict the flow of heated air and thermal radiation, providing the best possible thermal conditions. However, modifications can be made as needed, and further research is required to enhance the overall performance of ventilation hoods.

For the simulation, it was assumed that utilizing the values based on the perpendicular wind would be safe and conservative to begin designing such a hybrid ventilation system. If the direction of the wind changes or speeds differ, better placement or shape of openings, additional ventilation hoods, and mechanical ventilation units may be added for proper ventilation.

On the other hand, employing 500 mm diameter apertures yields around 5060 m³/min of natural ventilation, which is almost twice as much as needed. Therefore, larger diameter selection results in needless installation costs. In order to easily compare the three conventional systems of ventilation, a simple matrix is designed and is shown in Table 6.

Thermal performance of ETFE roofing systems depends on the amount of solar energy absorbed within the roof layers and how that affects the temperatures inside. High solar transmittance in ETFE membranes ensures the penetration of natural daylight, along with a reflection of a portion of infrared radiation, to minimize overheating. The advanced ETFE systems usually integrate coatings or multi-layer constructions that manage solar gain more effectively in terms of light transmission balanced by thermal insulation. The ETFE membranes have a high solar radiation transmittance of up to 0.8, which can be effectively modified by printing silver dots and by increasing thickness.

Innovative solutions have been explored to mitigate solar heat gain. Circulating water films on membrane roofs can significantly reduce surface temperatures and improve indoor thermal comfort [40]. Another approach involves installing solar shade films between ETFE layers, which can substantially reduce cooling demand. However, the optimal transmittance of these films must balance solar heat reduction with increased lighting energy needs [41]. These studies provide valuable insights into ETFE’s thermal behaviour and potential energy-saving strategies, contributing to more efficient building designs using this material.

Hybrid systems that integrate both mechanical and natural elements require specialized materials and labour due to the complexities involved, while the only drawback of the hybrid ventilation system is the difficulty in design, it can be seen that it excels in the rate of air exchange, thermal comfort, and energy efficiency compared to natural and mechanical ventilation systems. These systems consist of several sensors, control systems, and ventilation components that must be maintained and monitored to achieve an optimal level of performance. For example, false signals or misalignment between natural and mechanical components can result in inefficient system operation or system failure [42].

In addition, hybrid systems often require specialized designs for very specific climatic and structural conditions, which would compromise their scalability while demanding rigorous modelling during the design phase. These challenges demonstrate the necessity of planning and long-term cost–benefit analysis as conditions for making hybrid ventilation systems contenders for widespread acceptance (Principles of hybrid ventilation).

The cost of installation is also fairly reasonable, but the repair and maintenance costs and energy efficiency provide easy returns on the high initial installation cost. Hence, the hybrid ventilation system suited the subsurface cold storage facility the best because of the need for thermal comfort and low energy and power consumption. Since the main aim is to increase overall efficiency, the hybrid ventilation system is the best option.

## 8. Discussion and Conclusions

Biomimetic is the practice of emulating nature’s designs and processes to solve human problems. After including the discussed biomimetic features in the underground cold storage facility, the study showed significant long-term benefits over a conventional cold storage facility. Advancements have been made in underground cold storage, but several gaps remain in the literature. Most studies tend to deal with large-scale industrial storage rather than compact energy-sufficient solutions that find application in urban settings. While the application of some biomimetic features to underground thermal storage is understood in theory, there are no rigorous performance assessments conducted with tools like Revit-based simulations. The main gap in the current literature on underground cold storage is the lack of specific studies that apply biomimetic principles to energy-efficient cooling systems for underground conditions. Large volumes of research are available on the subject of biomimicry pertaining to architectural cooling or environmental systems, but very few have addressed the uniqueness of the underground challenges. Oftentimes, very few existing studies target a single biomimetic strategy like passive ventilation or heat dissipation without collating multi-faceted approaches that take multiple natural systems into account to address the complex underground storage needs.

Additionally, although a lot of theoretical work is available in the area of biomimetic designs, there is practically no implementation on the ground for underground energy-efficient systems. Most literature is abstract and not practically oriented towards the feasibility of such solutions with reference to construction and availability of materials. A key gap in existing research is the lack of real-world testing and practical designs adaptable to varying geographical and climatic conditions. Most studies tend to theorize without consideration of how these ideas are implemented in real environments. Another area lacking in research is the design of scalable, flexible solutions that can be tailored to various sites regarding local weather, materials, and costs. This study bridges the gap with a real-world testing-and-simulation approach in the creation of practical, adaptable solutions for underground cold storage. It is the purpose of the present study to fill the above-mentioned gaps by integrating multiple biomimetic strategies toward optimized underground cold storage, blending computational simulations and experimental validation to offer solutions that are both theoretically valid and practically feasible to construct and adapt to a variety of environments. Emphasis is placed on energy efficiency, operational cost savings, and sustainability in contrast to conventional active systems.

This will provide practical guidelines and bridge the gap between theory and real-world implementation.

The most important conclusions drawn from the study are that it has been efficient and savings concerning energy, time, land usage, and construction materials. The final design of the cold storage allows for a maximum storage capacity of 3500 MT—two cold storage chambers, each with a capacity of 1050 MT, and two pre-cooling rooms, each with a capacity of 700 MT.

The main reason why this method of construction is much more energy-efficient than traditional cold storage is that while constructing underground the temperature underground is already lower which eventually reduces the need for mechanical cooling, but taking, into consideration the deeper parts of the cold storage facility this paper discusses how to adopt the hybrid system as the natural airflow might not be enough in those places.

The heat losses are reduced to such a great extent and thermal efficiency is increased with the help of a composite wall which has taken inspiration from the Gentoo Penguin’s feathers. From these higher R-values and lower U-values, it reinforces the importance of insulation effectiveness in energy efficiency.

ETFE is lightweight which reduces the load-bearing capacity for the columns and beams. It is also an excellent material for the high transmittance of light. The use of a 3-layer ETFE cushion has wiped out the need for lighting fixtures. The usage of the concept of iceberg structures helped significantly increase the land use efficiency. The study revealed that by constructing an underground facility, at least 77% of the land would be saved, as constructing an entire 3500 MT cold storage facility at the surface would require at least two acres of land.

Using a system of hybrid ventilation underground can cut down on significant costs of mechanical ventilation. Rather than using a complete mechanical ventilation system, the hybrid system adopts several ventilation hoods that can ventilate naturally, along with a mechanical ventilation system that may be used only as a backup. This would increase the energy efficiency of the facility and, at the same time, be beneficial for the future in terms of economic savings. The hybrid ventilation system outshines a system of only natural or mechanical ventilation in terms of energy efficiency, rate of air exchange, thermal comfort, and repair and maintenance costs.

The storage facility is similar to the honeycomb structure. Each storage box is in the shape of a hexagon which increases the storage efficiency by 74%. The use of hexagonal boxes would also eliminate the possibility of any voids being formed in between the stacked boxes. Maximum produce can be stored in a small area in this way.

By implementing five biomimicry features, the facility will be able to achieve the target of being energy-efficient and reducing land usage. At the same time, it provides designers, engineers, and planners with a chance to look at underground construction positively. Although the initial cost may be high, there is a guarantee that the project will be able to save on recurring costs (electricity, repair, and maintenance). In this way, unnecessary land usage will be reduced, and there will be a scope for vertical construction in cities or towns where urban overcrowding is dominant. Innovative applications inspired by gentoo penguin feathers, iceberg structures, beehive shapes, and termite mounds seem promising but may face challenges of implementation on a global scale owing to climatic conditions. The heat-insulating capacity of feathers of penguins, for instance, might not be suitable in a hot climate, and the iceberg structure would require modifications to withstand places with extreme temperature variations or strong winds.

Natural ventilation-based termite mound-inspired passive cooling systems might be less effective in places with unsuitable wind patterns or certain soil compositions. Beehive-inspired buildings might also depend on locally available materials and construction methods that may not be universal across the world. The structures must be adjusted to be used in the local conditions in such a way that they will continue to work effectively, remain sustainable, and be economical in varied settings worldwide. Passive cooling systems based on termite mound designs may not be effective in climates that have no suitable wind patterns or soil configurations. These problems must be adequately considered when applying biomimetic concepts on an international scale to render them operational and sustainable. The approach’s effectiveness varies with climate and environmental factors, necessitating careful consideration of local conditions [43]. Despite these challenges, biomimicry presents opportunities for circular approaches, optimized resource use, and enhanced human-nature interactions in the built environment [44]. Ecosystem-level biomimicry, in particular, shows promise for creating regenerative architectural designs that go beyond sustainability to actively contribute to ecosystem health [45]. Successful implementation requires collaboration between biologists and engineers to overcome barriers and leverage nature’s solutions effectively [46,47].

The paper discussion is confined to a particular facet of design that emphasizes the adaption of energy components inspired by nature. Further studies can be focused on considering evaluating the natural temperature increase with depth in subterranean environments, identifying the ideal and maximum depths for underground constructions, many other design aspects and exploring other pertinent factors in food conservation, fire and safety needs to be explored. The future potential of biomimetics in civil engineering is promising as it marks the inception of developing sustainable and energy-efficient buildings inspired by nature, aligning effectively with the Sustainable Development Goals (SDGs).

## Figures and Tables

**Table 1:** Surface to Subsurface Ratio.

	Surface Facility	Subsurface Facility
Area	43.5 × 19 = 826.5 m^2^	43.5 × 45 = 1957.5 m^2^
Total height	4.5 m	9 m
Volume	826.5 × 4.5 = 3719.25 m^3^	1957.5 × 9 = 17617.5 m^3^
Ratio	=(Volume of the surface facility)/(Volume of the subsurface facility) = 3719.25/17617.5 = 1:4.7	

**Table 2:** U-value and G-value of conventional roofing materials Source: [35].

	6 mm Monolithic Glass	Double Glazing Unit	High Performance Double Glazing Unit	Regular Concrete Slab	2 Layer ETFE Cushion	3 Layer ETFE Cushion	4 Layer ETFE Cushion
U-Value (W/m^2^K)	5.9	2.8	2.0	1.3	2.9	1.9	1.4
G-Value	0.95	0.83	0.35	-	0.71–0.22 (with frit)	0.71–0.22 (with frit)	0.71–0.22 (with frit)

**Table 3:** Heat fluxes and temperatures for simplified glass and ETFE (for both cases direct solar transmission of 6% is assumed) Source: [36].

Parameters	Glass	ETFE
Longwave Radiative Grain (W/m^2^)	195	281
Convective Grain (W/m^2^)	73	56
Total Grain (W/m^2^)	328	334
External Layer Temperature (℃)	48	45
Interlayer Temperature (℃)	81	74
Internal layer Temperature (℃)	58	53

**Table 4:** Facility’s exterior walls components: Thicknesses and Thermal conductivities.

Walls	Thickness	Thermal Conductivity (ISO 8301)
Bagasse-cement composite	175 mm	1.03 W/mK
Thermal/air layer	100 mm	0.025 W/mK
Gypsum wall board	20 mm	0.65 W/mK
Metal stud layer	152 mm	0.052 W/mK
Concrete masonry	53 mm	1.3 W/mK

**Table 5:** Comparison of different structural combinations for the exterior walls.

Parameters	Structure A	Structure B	Structure C
Thickness	500 mm	500 mm	500 mm
No. of layers	5	5	5
R-Value	10.2 m^2^K/W	9.5 m^2^K/W	3.4 m^2^K/W
U-Value	0.098 W/m^2^K	0.105 W/m^2^K	0.294 W/m^2^K
Thermal Mass	33.77 kJ/K	34.05 kJ/K	55.64 kJ/K
Insulating Ability	Excellent	Very good	Poor
Seepage control	Excellent	Excellent	Poor
Heat Losses	Minimal	Minimal	Average
Internal Temperature	Comfortable	Comfortable	Not affected
Skill Required for instalment	High	High	Low

**Table 6:** Comparison of three conventional ventilation systems.

	Parameters Key: 0 = Poor; 5 = Average; 10 = Excellent						
Ventilation Systems	Cost to install	Cost for repair and maintenance	Energy Efficiency	Ease of design	Air Exchange Rate	Use of resources	Thermal Comfort
Natural	5	10	7	3	2	10	8
Mechanical	2	2	5	8	10	2	7
Hybrid	4	8	10	5	10	8	10

## Data Availability

All data are available within the manuscript.

## Supplementary Materials

The Supplementary material consists of “Developed Plans for the Biomimetic Subsurface Facility”. The supporting information can be downloaded at https://www.mdpi.com/article/10.3390/biomimetics10020122/s1. Figure S1 showing the developed plan of the Ground Floor and Figures S2 and S3 showing the developed plans of G-1 and G-2 levels, respectively.
