# Supplementary material for: Biomimicry-Based Design of Underground Cold Storage Facilities: Energy Efficiency and Sustainability
Source: Biomimetics (Basel). 2025 Feb 18;10(2):122. doi: 10.3390/biomimetics10020122 (PMC11853035; doi:10.3390/biomimetics10020122)
Supplement: Supplementary file 1 [file biomimetics-10-00122-s001.zip › biomimetics-3447732-supplementary_updated.pdf]

Supplementary material

Developed Plans for the Biomimetic Subsurface Facility

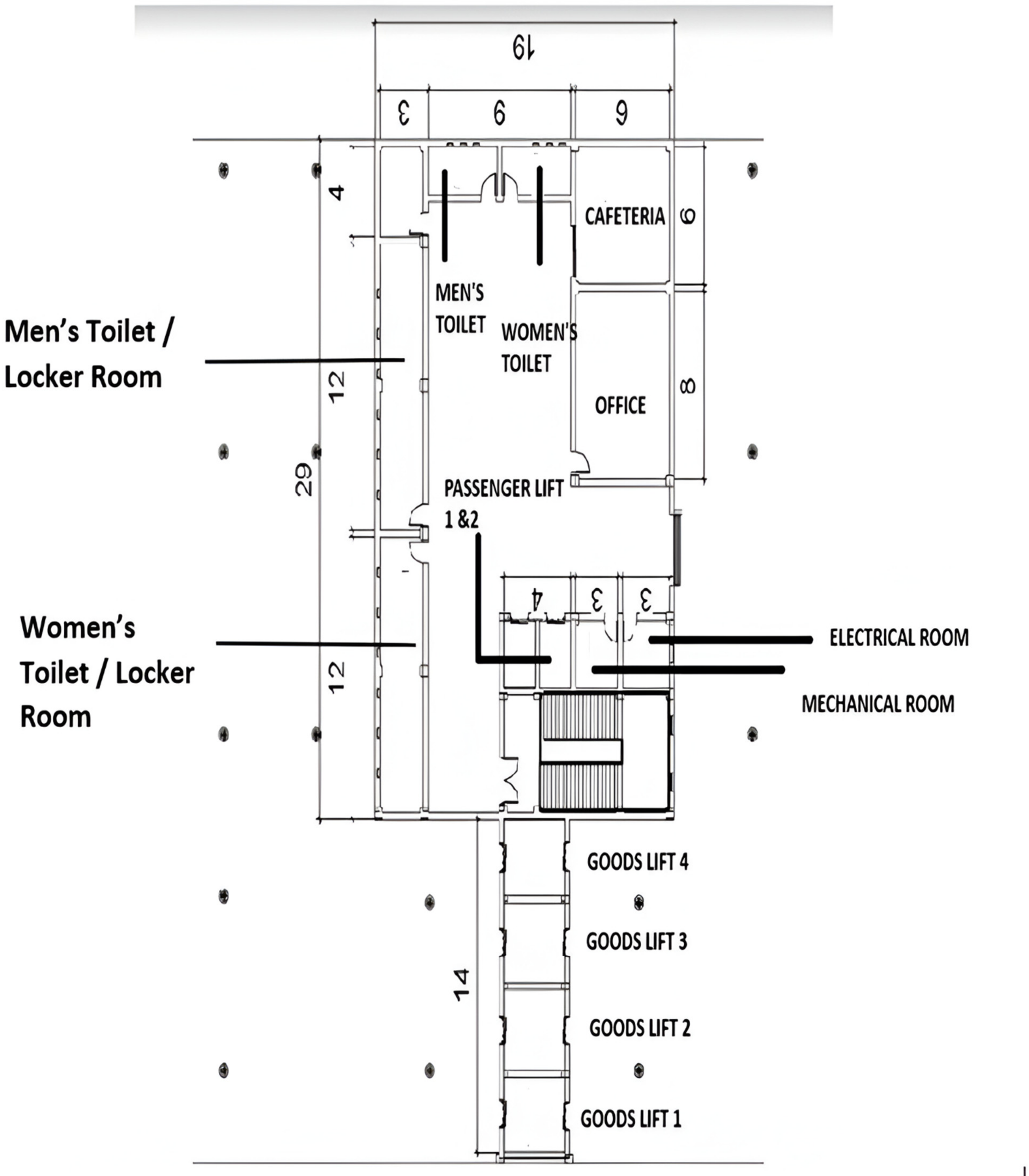

Figure S1 showing the developed plan of the Ground Floor

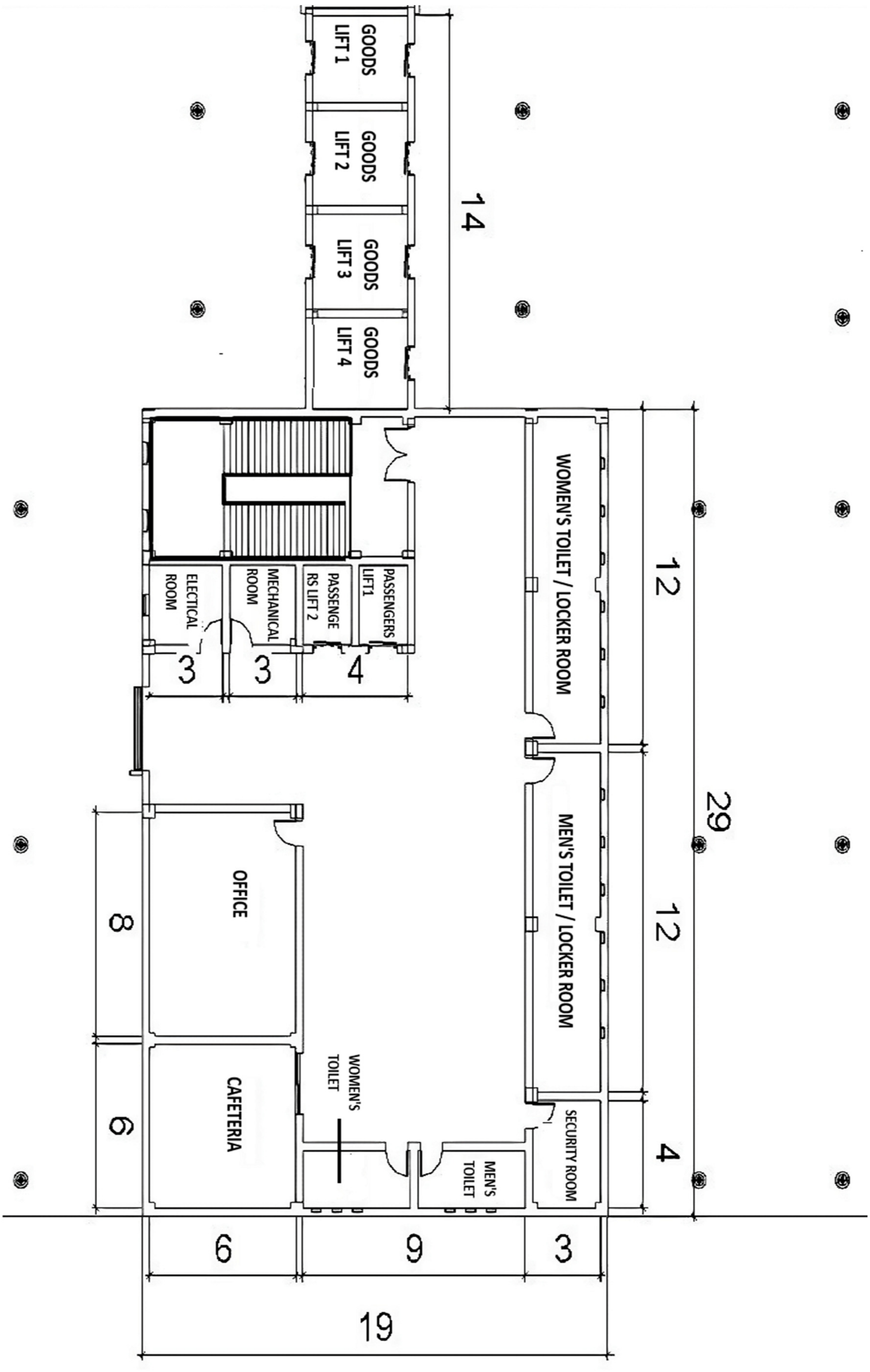

Figure S2 Showing the developed plans of G-1 level

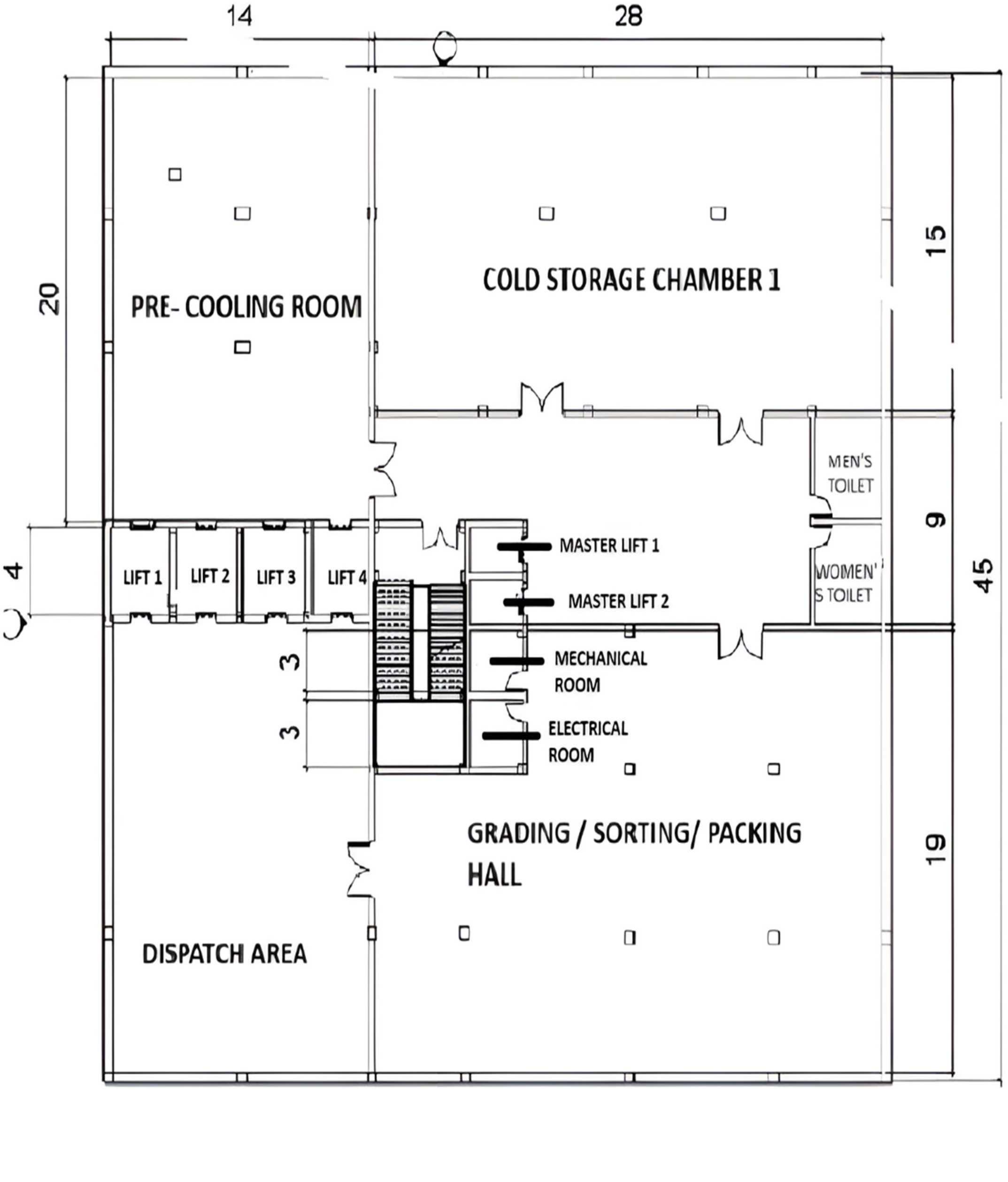

Figure S3 Showing the developed plans of G-2 level
